# 5-Nitro-2-(piperidin-1-yl)benzaldehyde

**DOI:** 10.1107/S1600536809043700

**Published:** 2009-10-28

**Authors:** A. J. N’Gouan, F. Mansilla-Koblavi, A. Timotou, A. Adjou, N. Ebby

**Affiliations:** a Laboratoire de Cristallographie et Physique Moléculaire, UFR SSMT, Université de Cocody 22 BP 582 Abidjan 22, Côte d’Ivoire; b Laboratoire de Chimie Organique, UFR SSMT, Université de Cocody 22 BP 582 Abidjan 22, Côte d’Ivoire

## Abstract

In the structure of the title compound, C_12_H_14_N_2_O_3_, the piperidine ring adopts a chair conformation and the aryl substitutent occupies an equatorial position.

## Related literature

For the toxicity of nitro­aromatics, see: Cronin *et al.* (1998 ▶); Shinoda *et al.* (1998 ▶). For piperidine ring conformations, see: Parkin *et al.* (2004 ▶). For ring conformational analysis, see: Cremer & Pople (1975 ▶). For reference bond lengths, see: Allen *et al.* (1987 ▶) and for bond angles, see; Codding & Kerr (1978 ▶).
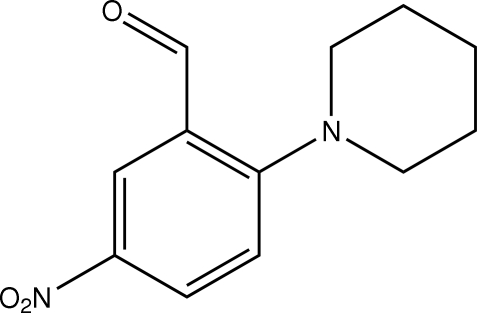


## Experimental

### 

#### Crystal data

C_12_H_14_N_2_O_3_
*M* *_r_* = 234.25
Triclinic, 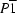

*a* = 5.686 (2) Å
*b* = 10.102 (5) Å
*c* = 10.221 (4) Å
α = 80.767 (2)°
β = 80.733 (3)°
γ = 86.034 (2)°
*V* = 571.4 (4) Å^3^
*Z* = 2
Mo *K*α radiation
μ = 0.10 mm^−1^
*T* = 295 K
0.30 × 0.25 × 0.25 mm

#### Data collection

Nonius KappaCCCD area-detector diffractometer
Absorption correction: none
11460 measured reflections
3280 independent reflections
2239 reflections with *I* > 2.0σ(*I*)
*R*_int_ = 0.03

#### Refinement

*R*[*F* ^2^ > 2σ(*F* ^2^)] = 0.045
*wR*(*F* ^2^) = 0.111
*S* = 1.02
2058 reflections
154 parameters
H-atom parameters constrained
Δρ_max_ = 0.16 e Å^−3^
Δρ_min_ = −0.15 e Å^−3^

### 

Data collection: *COLLECT* (Nonius, 1997 ▶); cell refinement: *DENZO*/*SCALEPACK* (Otwinowski & Minor, 1997 ▶); data reduction: *DENZO*/*SCALEPACK*; program(s) used to solve structure: *SIR2004* (Burla *et al.*, 2005 ▶); program(s) used to refine structure: *CRYSTALS* (Betteridge *et al.*, 2003 ▶); molecular graphics: *ORTEP-3* (Farrugia, 1997 ▶) and *PLATON* (Spek, 2009 ▶); software used to prepare material for publication: *CRYSTALS*.

## Supplementary Material

Crystal structure: contains datablocks I, global. DOI: 10.1107/S1600536809043700/ng2664sup1.cif

Structure factors: contains datablocks I. DOI: 10.1107/S1600536809043700/ng2664Isup2.hkl

Additional supplementary materials:  crystallographic information; 3D view; checkCIF report

## Figures and Tables

**Fig. 1.**
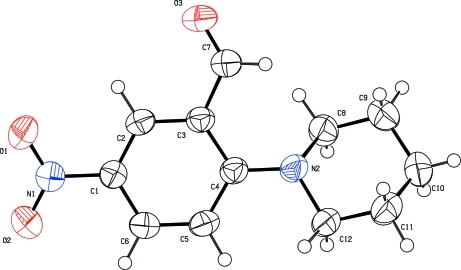
The title compound structure and atomic numbering scheme. Displacement ellipsoids are drawn at the 50% probability level. H atoms are shown as spheres of arbitary radius.

## supplementary crystallographic information

### Comment

Nitroaromatics are reactional intermediate compounds in chemical synthesis well known for their toxicity (Cronin *et al.*, 1998; Shinoda *et al.*,1998). Here we report the single-crystal X-ray determination of the title compound in order to have a best insight of its structure and then to undertake a study of its possible toxic activity. The molecular structure of this compound and its atomic labeling scheme are shown in Fig.1. In this one, the piperidine ring, (N2/C8/C9/C10/C11/C12), as previously reported (Parkin *et al.*, 2004), assumes a chair conformation, with the torsion angles mean value equal to 56.45 °, the puckering parameters (Cremer & Pople, 1975), being: Q = 0.5670 (17) Å, Phi = 365°, Theta = 1.59 (16)°. The C4 atom is in an equatorial position with respect to the piperidine ring. The system defined by (C1/C2/C3/C4/C5/C6), essentially planar, with a maximum deviation of 0.014 Å is an aromatic ring, according to the range of C—C bond lengths (1.3750 (19) Å to 1.4174 (17) Å) and C—C—C bond angles (117.61 (12) ° to 121.51 (12) °) (Peneloppe *et al.*, 1978). Values of selected bond lengths and angles are reported in table 1. The nitro group is confirmed throughout the N—O bond length characteristics, since distances d(O2—N1)=1.2195 (16)Å and d(O1—N1) =1.221 (16)Å are consistent with those encountered in the nitro group (Allen *et al.*, 1987). Besides d(C1—N1)=1.4553Å corresponds to a single bond length between an aromatic carbon and a nitrogen (Car—NO2). The bond length d(C7—O3)=1.2049 (18) Å, characterizes a normal double bond (CO) involved in an aldehyde function (Allen *et al.*, 1987).

### Experimental

[6.52 ml, (66 mmol)] of piperidine and [5.54 g, (66 mmol)] of sodium hydrogenocarbonate(NaHCO_3_) were added to [8 g, (43 mmol)] of distilled ethanol reflux during 24 h under shelter moister. After cooling to ambient temperature, the mixture was poured into 150 ml of dichloromethane then washed twice with 50 ml of water each time. After decantation, the organic layer was dried on magnesium sulfate, filtered and concentrated under reduced pressure. The residue was purifie by flash chromatography on silica gel using DCM/hexane(80/20)*v*/*v*. 8.8 g of the title compound were obtained with 85.51% yields. The melting point is 388k.

### Refinement

All the H atoms were found by difference fourier. their position and displacement parameters *U*_iso_(H) were refined to regularize their geometry(C—H in the range 0.94–0.99 Å) and *U*_iso_(H) (1.2 times *U*_eq_ of the parent atom), after which their positions were refined isotropically with riding constraints.

### Crystal data

**Table:**

C_12_H_14_N_2_O_3_	*Z* = 2
*M**_r_* = 234.25	*F*(000) = 248
Triclinic, *P*1	*D*_x_ = 1.362 Mg m^−^^3^
Hall symbol: -P 1	Melting point: 388 K
*a* = 5.686 (2) Å	Mo *K*α radiation, λ = 0.71073 Å
*b* = 10.102 (5) Å	Cell parameters from 3280 reflections
*c* = 10.221 (4) Å	θ = 2–30°
α = 80.767 (2)°	µ = 0.10 mm^−^^1^
β = 80.733 (3)°	*T* = 295 K
γ = 86.034 (2)°	Prism, orange
*V* = 571.4 (4) Å^3^	0.30 × 0.25 × 0.25 mm

### Data collection

**Table:**

Nonius KappaCCCD area detector diffractometer	*R*_int_ = 0.03
graphite	θ_max_ = 30.2°, θ_min_ = 2.0°
φ scans	*h* = 0→8
11460 measured reflections	*k* = −14→14
3280 independent reflections	*l* = −13→14
2239 reflections with *I* > 2.0σ(*I*)	

### Refinement

**Table:**

Refinement on *F*^2^	Primary atom site location: structure-invariant direct methods
Least-squares matrix: full	Hydrogen site location: inferred from neighbouring sites
*R*[*F*^2^ > 2σ(*F*^2^)] = 0.045	H-atom parameters constrained
*wR*(*F*^2^) = 0.111	Method = Modified Sheldrick *w* = 1/[σ^2^(*F*^2^) + ( 0.06*P*)^2^ + 0.1*P*] , where *P* = (max(*F*_o_^2^,0) + 2*F*_c_^2^)/3
*S* = 1.02	(Δ/σ)_max_ = 0.0004
2058 reflections	Δρ_max_ = 0.16 e Å^−^^3^
154 parameters	Δρ_min_ = −0.15 e Å^−^^3^
0 restraints	

### Special details

**Table:**

Refinement. We had 3280 independent reflections but 2058 reflections were used in the refinement, instead of 3280 because the refinement was carried out under conditions I > 3σ(I).

### Fractional atomic coordinates and isotropic or equivalent isotropic displacement parameters (Å^2^)

**Table:**

	*x*	*y*	*z*	*U*_iso_*/*U*_eq_	
N1	−0.2540 (2)	−0.19297 (12)	0.65442 (13)	0.0538	
N2	0.1530 (2)	0.26552 (11)	0.75678 (11)	0.0494	
C1	−0.1479 (2)	−0.07454 (13)	0.68028 (13)	0.0446	
C2	0.0235 (2)	−0.08957 (12)	0.76296 (12)	0.0437	
C3	0.1255 (2)	0.02260 (12)	0.78871 (12)	0.0421	
C4	0.0534 (2)	0.15293 (12)	0.73040 (12)	0.0428	
C5	−0.1174 (3)	0.16295 (13)	0.64329 (14)	0.0514	
C6	−0.2181 (3)	0.05102 (14)	0.61906 (14)	0.0516	
C7	0.3268 (3)	−0.00219 (15)	0.86587 (14)	0.0530	
C8	0.1313 (3)	0.28678 (15)	0.89744 (13)	0.0539	
C9	0.3194 (3)	0.37797 (17)	0.91473 (16)	0.0665	
C10	0.3053 (4)	0.51042 (17)	0.82255 (17)	0.0676	
C11	0.3178 (3)	0.48655 (15)	0.67935 (16)	0.0630	
C12	0.1293 (3)	0.39255 (14)	0.66607 (15)	0.0573	
O1	−0.1742 (2)	−0.30357 (10)	0.69841 (14)	0.0772	
O2	−0.4183 (2)	−0.17831 (12)	0.58925 (14)	0.0796	
O3	0.3735 (2)	−0.10961 (12)	0.92869 (13)	0.0761	
H2	0.0775	−0.1770	0.8003	0.0524*	
H5	−0.1691	0.2482	0.6013	0.0617*	
H6	−0.3366	0.0604	0.5608	0.0619*	
H7	0.4264	0.0706	0.8620	0.0636*	
H81	0.1423	0.1992	0.9549	0.0647*	
H82	−0.0258	0.3295	0.9232	0.0647*	
H91	0.3019	0.3901	1.0089	0.0798*	
H92	0.4712	0.3336	0.8926	0.0798*	
H101	0.4289	0.5645	0.8298	0.0812*	
H102	0.1507	0.5572	0.8515	0.0812*	
H111	0.2952	0.5712	0.6206	0.0756*	
H112	0.4750	0.4465	0.6503	0.0756*	
H121	−0.0316	0.4328	0.6880	0.0688*	
H122	0.1462	0.3734	0.5738	0.0688*	

### Atomic displacement parameters (Å^2^)

**Table:**

	*U*^11^	*U*^22^	*U*^33^	*U*^12^	*U*^13^	*U*^23^
N1	0.0571 (7)	0.0479 (7)	0.0585 (7)	−0.0037 (5)	−0.0156 (6)	−0.0069 (5)
N2	0.0716 (8)	0.0387 (5)	0.0387 (5)	−0.0033 (5)	−0.0132 (5)	−0.0032 (4)
C1	0.0483 (7)	0.0414 (7)	0.0447 (7)	−0.0017 (5)	−0.0089 (5)	−0.0067 (5)
C2	0.0490 (7)	0.0396 (6)	0.0400 (6)	0.0018 (5)	−0.0067 (5)	−0.0001 (5)
C3	0.0477 (7)	0.0415 (6)	0.0365 (6)	−0.0001 (5)	−0.0085 (5)	−0.0023 (5)
C4	0.0519 (7)	0.0396 (6)	0.0361 (6)	0.0001 (5)	−0.0066 (5)	−0.0043 (5)
C5	0.0636 (9)	0.0401 (7)	0.0508 (7)	0.0056 (6)	−0.0187 (6)	−0.0011 (5)
C6	0.0570 (8)	0.0492 (7)	0.0510 (7)	0.0025 (6)	−0.0204 (6)	−0.0044 (6)
C7	0.0565 (8)	0.0502 (8)	0.0527 (8)	−0.0027 (6)	−0.0168 (6)	−0.0006 (6)
C8	0.0693 (9)	0.0528 (8)	0.0409 (7)	−0.0078 (7)	−0.0078 (6)	−0.0093 (6)
C9	0.0804 (11)	0.0674 (10)	0.0576 (9)	−0.0135 (8)	−0.0267 (8)	−0.0066 (8)
C10	0.0807 (11)	0.0571 (9)	0.0718 (10)	−0.0192 (8)	−0.0251 (9)	−0.0094 (8)
C11	0.0770 (11)	0.0475 (8)	0.0622 (9)	−0.0086 (7)	−0.0112 (8)	0.0018 (7)
C12	0.0848 (11)	0.0394 (7)	0.0501 (8)	−0.0030 (7)	−0.0215 (7)	−0.0026 (6)
O1	0.0870 (8)	0.0420 (6)	0.1096 (10)	−0.0025 (5)	−0.0414 (7)	−0.0056 (6)
O2	0.0848 (8)	0.0650 (7)	0.1011 (10)	−0.0102 (6)	−0.0519 (8)	−0.0082 (7)
O3	0.0848 (8)	0.0595 (7)	0.0874 (9)	−0.0029 (6)	−0.0478 (7)	0.0128 (6)

### Geometric parameters (Å, °)

**Table:**

N1—O1	1.2210 (16)	C1—C6	1.3839 (19)
N1—C1	1.4553 (18)	C6—H61	0.959
N1—O2	1.2195 (16)	C8—C9	1.507 (2)
C3—C4	1.4174 (17)	C8—H81	0.984
C3—C2	1.3878 (19)	C8—H82	0.980
C3—C7	1.4773 (19)	C12—C11	1.516 (2)
C4—N2	1.3868 (17)	C12—H122	0.981
C4—C5	1.4081 (19)	C12—H121	0.982
N2—C8	1.4720 (18)	C9—C10	1.513 (2)
N2—C12	1.4694 (17)	C9—H92	0.956
C5—C6	1.376 (2)	C9—H91	0.978
C5—H51	0.949	C11—C10	1.511 (2)
C2—C1	1.3750 (19)	C11—H112	0.977
C2—H21	0.955	C11—H111	0.977
C7—O3	1.2049 (18)	C10—H101	0.939
C7—H71	0.951	C10—H102	0.993
			
O1—N1—C1	118.62 (12)	C9—C8—H81	111.4
O1—N1—O2	122.42 (13)	N2—C8—H82	108.6
C1—N1—O2	118.96 (12)	C9—C8—H82	108.4
C4—C3—C2	120.29 (12)	H81—C8—H82	108.4
C4—C3—C7	122.63 (12)	N2—C12—C11	109.92 (13)
C2—C3—C7	116.75 (12)	N2—C12—H122	108.8
C3—C4—N2	120.58 (12)	C11—C12—H122	110.7
C3—C4—C5	117.61 (12)	N2—C12—H121	109.0
N2—C4—C5	121.79 (12)	C11—C12—H121	110.9
C4—N2—C8	118.01 (11)	H122—C12—H121	107.5
C4—N2—C12	118.40 (11)	C8—C9—C10	110.77 (14)
C8—N2—C12	111.78 (11)	C8—C9—H92	107.4
C4—C5—C6	121.51 (12)	C10—C9—H92	109.5
C4—C5—H51	120.3	C8—C9—H91	109.3
C6—C5—H51	118.1	C10—C9—H91	112.0
C3—C2—C1	119.98 (12)	H92—C9—H91	107.7
C3—C2—H21	119.5	C12—C11—C10	111.64 (13)
C1—C2—H21	120.4	C12—C11—H112	108.8
C3—C7—O3	123.42 (14)	C10—C11—H112	108.6
C3—C7—H71	117.1	C12—C11—H111	108.5
O3—C7—H71	119.4	C10—C11—H111	110.5
N1—C1—C2	119.43 (12)	H112—C11—H111	108.8
N1—C1—C6	119.35 (12)	C9—C10—C11	110.19 (14)
C2—C1—C6	121.20 (12)	C9—C10—H101	110.3
C1—C6—C5	119.35 (13)	C11—C10—H101	110.4
C1—C6—H61	120.6	C9—C10—H102	107.9
C5—C6—H61	120.1	C11—C10—H102	109.6
N2—C8—C9	110.88 (12)	H101—C10—H102	108.3
N2—C8—H81	109.1		
			
C12—N2—C8—C9	−58.84 (17)	C8—C9—C10—C11	−54.06 (18)
C8—N2—C12—C11	58.21 (16)	C9—C10—C11—C12	54.45 (18)
N2—C8—C9—C10	56.24 (17)	C10—C11—C12—N2	−56.22 (17)
